# Application of Circumferential Compression Device (Binder) in Pelvic Injuries: Room for Improvement

**DOI:** 10.5811/westjem.2016.7.30057

**Published:** 2016-10-20

**Authors:** Rahul Vaidya, Matthew Roth, Bradley Zarling, Sarah Zhang, Christopher Walsh, Jessica Macsuga, John Swartz

**Affiliations:** * Wayne State University School of Medicine, Department of Orthopaedic Surgery and Emergency Medicine, Detroit, Michigan; † Detroit Medical Center, Department of Emergency Medicine, Detroit, Michigan

## Abstract

**Introduction:**

The use of a noninvasive pelvic circumferential compression device (PCCD) to achieve pelvic stabilization by both decreasing pelvic volume and limiting inter-fragmentary motion has become commonplace, and is a well-established component of Advanced Trauma Life Support (ATLS) protocol in the treatment of pelvic ring injuries. The purpose of this study was to evaluate the following: 1) how consistently a PCCD was placed on patients who arrived at our hospital with unstable pelvic ring injuries; 2) if they were placed in a timely manner; and 3) if hemodynamic instability influenced their use.

**Methods:**

We performed an institutional review board-approved retrospective study on 112 consecutive unstable pelvic ring injuries, managed over a two-year period at our Level I trauma center. Our hospital electronic medical records were used to review EMT, physician, nurses’, operative notes and radiographic images, to obtain information on the injury and PCCD application. The injuries were classified by an orthopaedic trauma surgeon and a senior orthopaedic resident. Proper application of a pelvic binder using a sheet is demonstrated.

**Results:**

Only 47% of unstable pelvic fractures received PCCD placement, despite being the standard of care according to ATLS. Lateral compression mechanism pelvic injuries received PCCDs in 33% of cases, while anterior posterior compression (APC) and vertical shear (VS) injuries had applications in 63% of cases. Most of these PCCD devices were applied after imaging (72%). Hemodynamic instability did not influence PCCD application.

**Conclusion:**

PCCD placement was missed in many (37%) of APC and VS mechanism injuries, where their application could have been critical to providing stability. Furthermore, to provide rapid stability, pelvic circumferential compression devices should be applied after secondary examination, rather than after receiving imaging results. Better education on timing and technique of PCCD placement at our institution is required to improve treatment of pelvic ring injuries.

## INTRODUCTION

**Figure 1:**
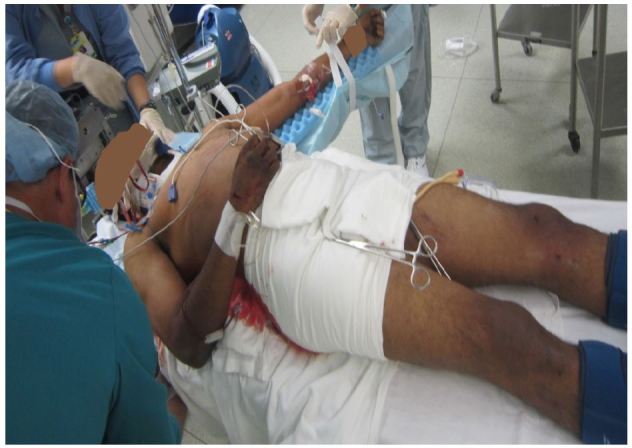
Use of a noninvasive pelvic circumferential compression device (PCCD) has become commonplace, and has become a well-established component of ATLS protocol.

**Figure 2:**
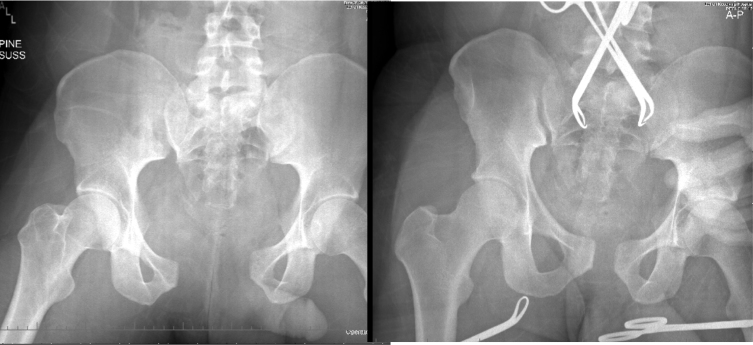
Both commercial binders and traditional sheeting techniques seem to be effective at reducing pelvic volume. A: Before application of pelvic binder, B: After application of pelvic binder. Note significant reduction in displacement with smaller pelvis volume.

Pelvic ring injuries carry a high burden of mortality and morbidity.^1^ Life-threatening retroperitoneal hemorrhage can occur due to shearing of pelvic vessels as well as bleeding from fractured bone ends,^2^ contributing to morbidity. However, it is postulated that early pelvic stabilization may help prevent exsanguination by decreasing pelvic volume and limiting inter-fragmentary motion, permitting stable clot formation. Use of a noninvasive pelvic circumferential compression device (PCCD) to achieve this effect has become commonplace, and has become a well-established component of Advanced Trauma Life Support (ATLS) protocol^3^ (Figure 1). Both commercial binders and traditional sheeting techniques seem to be effective in reducing pelvic volume^4^,^5^ (Figure 2). Pelvic binders are used not only at major trauma centers, but in prehospital and pre-transfer settings.^6^ Pelvic fractures classification has an important role in the decision of whether or not to place a PCCD. The Young and Burgess classification looks at pelvic fractures in terms of the mechanism of injury: anterior posterior compression (APC, open book), lateral compression (LC), vertical shear (VS) or combined mechanism (CM). Stable injuries include APC1 and LC1, while LC2, LC3, APC2, APC3, VS and CM are unstable injures.^7^,^8^,^9^ In the Young and Burgess classification, increasing numbers signify increasing severity of pelvic ring injury (Video 1). PCCDs are indicated for APC, VS, CM and LC3 lateral compression mechanisms. Their use in other LC injuries is not helpful, but the drawbacks are few if any.^10^,^11^,^12^,^13^,^14^,^15^

The purpose of this study was to evaluate 1) how consistently a PCCD was placed on patients who arrived at our hospital with unstable pelvic ring injuries; 2) if they were placed in a timely manner; and 3) if hemodynamic instability influenced their use.

## METHODS

We used an institional review board approved-retrospective study using data collected from our Level I trauma center. Detroit Receiving Hospital (Detroit Medical Centre/Wayne State University) is an urban hospital with 120,000 annual emergency department (ED) visits, and is noted as being America’s first verified Level I trauma center. The hospital’s protocol for care of pelvic ring injuries included standard ATLS guidelines. A primary survey is followed by a secondary survey that includes physical assessment of pelvic stability, and upon detection of an unstable pelvic injury, a clamped sheet or PCCD is placed. The trauma codes are run either by general surgery or the emergency physicians, and orthopaedic residents or staff act as consultants during trauma codes and are summoned to the trauma bay. All patients get an initial anterior-posterior trauma pelvis radiograph, and most trauma codes get a computed tomography (CT) of the abdomen and pelvis as well. If the patient was transferred with a PCCD in place, it was left in place until definitive management was performed.

This study included 112 consecutive patients with unstable pelvic ring injuries that were managed at our institution over a two-year period; we excluded patients with stable injuries from the study. Patients ranged in age from 18 to 86 years, with an average age of 41+15 (median 41) years. Of the patients, 35 (31%) were women and 77 (69%) were men. Every patient included in the study underwent surgical fixation.

We reviewed the chart, ED attending, resident and nurses’ notes, radiographss and CTs. Injuries were classified by an orthopaedic traumatologist and a senior orthopaedic resident. In the case of discrepancy the case was discussed and a consensus reached. We noted when and if a PCCD was applied, whether it was placed prior to x-rays, prior to or after CT or not at all. We also recorded the patients’vitals upon arrival, and their ATLS hemorrhage class. The ATLS hemorrhage class is based on heart rate (HR), blood pressure (BP), respiratory rate, mental status and urinary output. As all patents do not fall strictly into categories (mental status was not clearly recorded for all patients and urinary output changes during resuscitation), we based our classification on HR, BP, and any other information we could garner from the ED notes including transfusion. Patients were thus classified as class 1 to 4 hemorrhage but for comparison between groups we listed the patient as hemodynamically stable or unstable. Class 1 was felt to be stable and Classes 2, 3 and 4 were considered unstable.

All patients presenting with pelvic fractures should have had a PCCD placed according to ATLS protocol, which recommends PCCD or sheet placement in unstable pelvic fractures after physical pelvic examination, before interpretation of radiographic results. However, if an LC mechanism was identified by the physician, not placing a PCCD would not have been harmful to the care of the patient. Thus, we separated the cases by mechanism into two groups APC, VS and CM (group 1) and LC (group 2).

## RESULTS

We classified patients’ injuries according to the Young and Burgess classification scheme, with their vital signs and hemorrhage class, hemodynamically stable or unstable (Tables 1, 2, and 3).

Pelvic circumferential compression devices were used in 47% (55/112) of the patients. Patients who we identified as having either an APC or VS type injury comprised 69% (38/55) of the patients treated with a PCCD. Conversely, 31% (17/55) of patients had PCCDs placed for partial or complete LC injuries. Of the 57 pelvic ring injuries not managed with a PCCD, 40% (23/57) had an APC or VS mechanism, and 60% (34/57) had an LC mechanism (Table 1). We missed placing a PCCD in 38% of unstable APC or VS (23/61) mechanism patients and 67% (34/51) of unstable LC mechanisms.

### Timing of PCCD Placement

Application of the PCCD occurred prior to a radiograph at our institution in six patients; 38 patients had the PCCD placed between taking an AP pelvic radiograph and the CT. Four patients had PCCDs placed after the CT scan and seven patients were transferred to our hospital with a PCCD prior to arrival. As all patients had unstable pelvic injuries in this series, it is safe to say that that we picked up an unstable pelvic injury from the secondary survey and applied a PCCD in only 6/112 patients. The unstable injury was recognized and treated with a PCCD after radiograph in 38/106 patients and after CT in 4/68 patients who were eligible for PCCD placement.

### Vitals Signs and Hemorrhage Class

We further assessed if PCCD placement was influenced by hemodynamic instability at presentation (Table 2 and Table 3). Patients were classified by hemodynamic shock class, with Class 1 being stable and Classes 2, 3 and 4 signifying hemodynamically unstable patients. Classes 2, 3 and 4 patients were grouped together to form a “hemodynamically unstable” group, for comparison with the Class 1 patients, who were labeled “stable” (Table 2, 3). Thirty patients classified with hemodynamic instability had a PCCD placed, and there were 25 patients with hemodynamic instability without PCCD placement placed. These groupings were then used in a Student’s t-test, comparing the distribution of stable and unstable fractures for patients who had pelvic binders applied and those who did not. While the patients without binders tended to have more stable injuries, the t-test showed that there was no statistically significant difference between the patients with and without PCCDs (p=.301). Another t-test was performed comparing the groups with and without binders, but by discrete hemodynamic shock category, rather than just stable and unstable injuries. While this showed a slightly improved p-value, it still lacked significance (p=.247), indicating no significant relationship between hemodynamic shock class and the choice of PCCD placement with respect to our data.

## DISCUSSION

**Figure 3:**
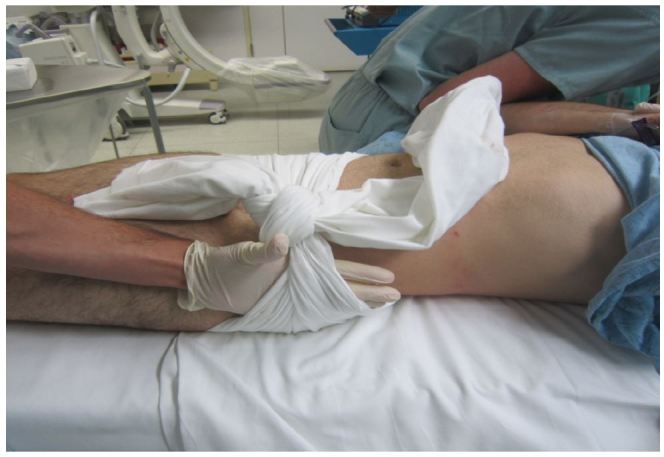
Poorly applied pelvic circumferential compression device: too loose, too low and should not be tied.

**Figure 4:**
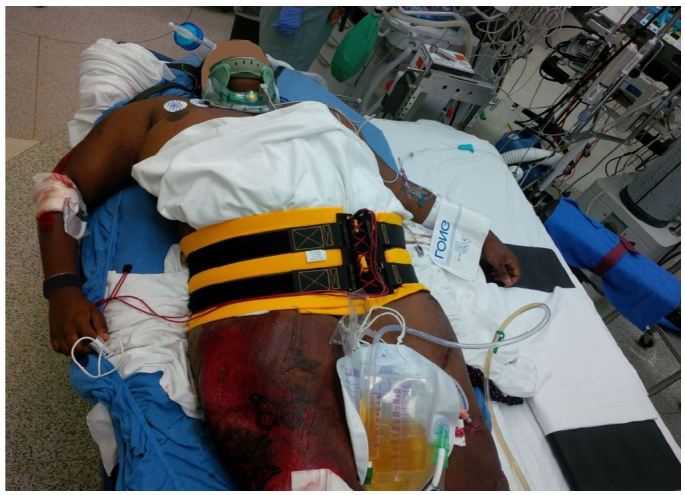
Poorly applied pelvic circumferential compression: It is too high on the belly and should be at the level of the greater trochanter.

The use of PCCDs in the treatment of pelvic injuries has become the standard of care,^3^ particularly in APC and VS injury mechanisms. Their benefits include lifesaving hemorrhage control,^5^,^10^,^11^ decreased mortality,^10^ reduced transfusion requirement,^5^,^10^,^11^ pelvic fracture reduction/stabilization,^5^,^10^,^11^,^15^, length of hospital stay,^5^,^10^,^11^ pain control, low risk, non invasive, easy to apply and cheap.^5^,^10^,^11^,^12^,^13^,^14^,^15^ In patients who were transferred to another institution with a pelvic ring injury, applying a PCCD led to significantly decreased transfusion requirements whether they were hemodynamically stable or unstable prior to transfer.^5^ The drawbacks of using PCCD are few, if any, even with LC mechanisms.^10^,^11^,^12^,^13^,^14^,^15^ They allow adequate exposure if laparotomy or angiography are indicated.^6^ PCCDs are more effective if placed accurately at the level of the greater trochanters and not higher on the abdomen, which is the most common error^16^ (Figure 3 and Figure 4, Video 2). Although there are several different types of commercially available binders, there is no evidence to show superiority of one particular model even over pelvic sheets, which are commonly used.^5^ There are complications associated with their use, such as pressure sores, tissue necrosis and nerve palsy,^7^ especially if they are left on for a prolonged period of time. Pelvic binders may mask the “severity” of the pelvic injury on CTs, particularly APC patterns.^17^ It is rare to completely hide any injury, but it does happen.^17^,^18^ This is not a reason to avoid PCCD usage but an example of how efficient they are at accomplishing their goal. For the trauma team, one should be aware that a CT with a PCCD placed without prior imaging may not be diagnostic of the injury.^17^ For the treating surgeon, a fluoroscopic exam under anesthesia in a controlled environment (the operating room) is an important adjunct in this situation.^17^ We don’t recommend removing the PCCD to do a radiograph in a hemodynamically unstable patient. Important limitations of pelvic binders are that they do not control VS fractures and do not stop arterial bleeding; therefore, access to provide embolization is vital. It is important to place binders expediently in patents with pelvic hemorrhage, and the reason for this study. We did not find any previous studies looking at the timing of PCCD placement in ED patients in relationship to radiographs and CTs, except one looking at how well PCCDs reduce and can mask pelvic injuries.^17^

We found that despite ATLS teaching of PCCD placement, on any unstable pelvic injury at our institution we only accomplished this in 47% (55/112) of such cases in this series. When we looked at just APC or VS injuries, the rate of use improved to 63% of cases (38/61). This still left a significant number of patients (37% [23/61]) without a PCCD placed for an APC or VS mechanism.

For LC mechanisms where the indication for a PCCD is questionable except in the LC3 mechanism we found that PCCDs were placed in 33% of cases. The fear of using PCCDs in LC mechanisms is that they will over-compress the fracture and could lead to further injury, and so some controversy exists with these injuries.^12^ The general feeling is that a PCCD should be placed in any unstable mechanism so that emergency physicians or early responders do not have to make any decisions based on radiographs or the CT. If that is the case, we missed 67% of cases of LC injuries where a PCCD should have been placed. However, many emergency physicians, general surgeons and residents can read radiographs, classify pelvic injuries, and may have elected not to place the binder in the LC mechanisms. Nonetheless, according to ATLS procedure, pelvic binder placement should occur before radiograph interpretation.

We found that when PCCD devices were placed, they were done so after imaging, either after radiograph and before CT (38), or after the CT(4). Only six patients had the PCCD placed after clinical examination, and prior to radiograph. Thus, we may need to reinforce that an exam of the pelvis should be done in the secondary survey and that if a pelvic injury is suspected, a PCCD should be placed immediately. We are not sure if our staff missed identifying the injury on exam of the pelvis, were hesitant to place a binder until after imaging, or were uncomfortable placing a PCCD.

The quality of the binder placement was variable. We were not able to rate every case of PCCD application; we did find that many were placed high on the ilium rather than over the greater trochanters, which is a common error.^16^ We did not notice any specific complications as most of them were removed within six to eight hours.

We found that hemodynamic instability was not a great predictor of PCCD placement in our patients.

## LIMITATIONS

This study was limited by its retrospective and observational design, as well as sample size. However, we were able to get an idea of how often PCCDs were applied when indicated at our institution. We will continue to educate the frontline physicians in this apparatus, how to place it and the timing of application (Video 1,2). Others have also noted variability in knowledge, use and application of PCCDs.^5^ The authors acknowledge that no formal study of inter-observer agreement was performed for the radiographic classification of the injuries, but diagnosis were discussed when there was a discrepancy and a consensus was reached. We also did not ascertain whether placement of a PCCD and the timing of PCCD placement affected patient outcomes. Our numbers were low for this type of comparison and other groups have studied this, as mentioned in the discussion. 5,10,11,12,13,14,15

## CONCLUSION

The current ATLS teaching is placing a PCCD expediently with suspected pelvic instability. At our institution we missed application of a PCCD in 37% of APC/VS mechanisms and 67% of LC mechanisms (which may still have some controversy). We could be more effective at diagnosing these injuries during our secondary survey instead of waiting for the plain radiograph or CT. There is a need to educate and reeducate the frontline providers on the timely placement of PCCDs.

## Figures and Tables

**Video 1:** Identifying pelvic ring injuries and the Young and Burgess classification.

**Video 2:** Application of a Pelvic Binder using a common sheet.

**Table 1:** Mechanism of injury vs binder placement.

Mechanism of unstable pelvic injuries	PCCD placed	PCCD not placed	Total of PCCD placed and not placed
APC/VS	38	23	61
LC	17	34	51
APC/VS and LC Total	55	57	112

*APC,* anterior posterior compression; *VS,* vertical shear; LC, lateral compression; *PCCD,* pelvic circumferential compression device.

**Table 2:** Young and Burgess (Y and B) classification vitals signs and shock class (with binder). *APC,* anterior posterior compression; *LC,* lateral compression.

Patient binder placement	Y and B class	Pulse on arrival	BP	Shock class hemodynamically stable/unstable
1	APC3	70	108/50	1 Stable
2	APC2	117	117/68	2 Unstable
3	APC3	78	102/80	1 Stable
4	APC2	78	156/95	1 Stable
5	APC3	80	90/60	2 Unstable
6	APC2	121	80/52	3 Unstable
7	LC3	120	60/30	4 Unstable
8	APC2	122	147/102	2 Unstable
9	APC2	92	124/78	1 Stable
10	LC3	83	105/56	1 Stable
11	APC3	98	148/108	1 Stable
12	LC3	107	119/90	2 Unstable
13	APC2	83	132/82	1 Stable
14	LC3	80	157/86	1 Stable
15	APC2	80	125/65	1 Stable
16	LC2	86	110/80	1 Stable
17	APC3	100	155/96	2 Unstable
18	APC3	70	90/58	2 Unstable
19	APC3	90	120/86	1 Stable
20	APC2	105	114/68	2 Unstable
21	LC3	92	134/74	1 Stable
22	APC3	130	60/	4 Unstable
23	APC3	128	103/86	3 Unstable
24	APC2	106	96/66	2 Unstable
25	LC3	70	135/90	1 Stable
26	LC3	109	60/30	4 Unstable
27	LC3	145	96/66	3 Unstable
28	LC2	76	130/90	1 Stable
29	APC3	50	105/60	1 Stable
30	LC2	120	131/78	2 Unstable
31	LC2	71	130/90	1 Stable
32	APC3	100	103/59	2 Unstable
33	APC3	87	130/68	1 Stable
34	APC3	90	209/188	1 Stable
35	APC3	86	93/64	1 Stable
36	APC3	87	172/94	1 Stable
37	LC1	121	122/71	2 Unstable
38	LC3	92	116/74	1 Stable
39	APC3	125	98/47	3 Unstable
40	APC3	137	170/130	2 Unstable
41	APC3	93	133/100	1 Stable
42	APC3	113	110/80	2 Unstable
43	APC3	138	139/70	2 Unstable
44	APC3	120	124/85	2 Unstable
45	LC1	111	139/95	2 Unstable
46	APC2	67	213/114	1 Stable
47	LC3	105	199/85	2 Unstable
48	APC3	147	97/71	3 Unstable
49	APC2	86	140/70	1 Stable
50	APC3	120	70/50	4 Unstable
51	APC2	65	137/70	1 Stable
52	APC3	101	132/71	2 Unstable
53	LC2	109	101/75	2 Unstable
54	APC2	150	120/70	3 Unstable
55	APC3	140	90/60	3 Unstable

*APC,* anterior posterior compression; *LC,* lateral compression.

**Table 3:** Young and Burgess (Y and B) classification vitals signs and shock class (without binder). *APC,* anterior posterior compression; *LC,* lateral compression.

Patient no binder	Y and B class	Pulse on arrival	BP	Shock class hemodynamically stable/unstable
1	APC3	98	158/107	1 Stable
2	APC3	86	114/54	1 Stable
3	LC2	106	87/42	3 Unstable
4	LC3	109	147/112	2 Unstable
5	LC3	119	152/82	2 Unstable
6	APC2	154	98/58	3 Unstable
7	LC3	66	122/86	1 Stable
8	LC3	97	110/60	1 Stable
9	LC3	76	104/43	1 Stable
10	APC2	86	114/54	1 Stable
11	LC3	133	91/47	3 Unstable
12	APC2	106	96/50	2 Unstable
13	LC2	113	117/78	2 Unstable
14	LC2 BILAT	105	130/94	2 Unstable
15	LC2	96	178/100	1 Stable
16	APC3	81	142/96	1 Stable
17	LC2	90	93/70	2 Unstable
18	LC3	94	97/49	2 Unstable
19	LC2	94	117/85	1 Stable
20	LC2	94	97/49	2 Unstable
21	LC3	140	90/50	3 Unstable
22	LC2	140	68/43	4 Unstable
23	LC2	108	121/85	2 Unstable
24	LC2	77	90/68	1 Stable
25	LC2	82	103/53	1 Stable
26	APC3	157	53/52	4 Unstable
27	APC2	84	130/75	1 Stable
28	LC2	85	127/83	1 Stable
29	LC2	87	112/82	1 Stable
30	LC3	84	144/107	1 Stable
31	APC3	106	84/50	2 Unstable
32	APC3	114	147/120	2 Unstable
33	APC3	90	140/180	1 Stable
34	LC2	77	130/73	1 Stable
35	LC2	87	133/92	1 Stable
36	APC2	64	121/78	1 Stable
37	LC2	86	100/60	1 Stable
38	LC2	86	104/63	1 Stable
39	APC2	68	110/72	1 Stable
40	LC2	125	142/95	2 Unstable
41	LC2	81	118/82	1 Stable
42	LC2	104	108/68	2 Stable
43	LC2	150	103/81	3 Unstable
44	APC2	85	159/107	1 Stable
45	APC2	105	100/75	2 Unstable
46	APC2	67	160/83	1 Stable
47	LC2	74	114/85	1 Stable
48	APC3	105	156/92	2 Unstable
49	APC2	79	138/97	1 Stable
50	LC2	90	152/87	1 Stable
51	LC2	110	148/76	2 Unstable
52	APC3	126	1037/97	2 Unstable
53	APC3	70	120/75	1 Stable
54	LC2	120	90/60	3 Unstable
55	APC2	92	134/78	1 Stable
56	APC3	98	137/68	1 Stable
57	APC3	99	140/70	1 Stable

*APC,* anterior posterior compression; *LC,* lateral compression.

## References

[1] Gabbe BJ, de Steiger R, Esser M (2011). Predictors of mortality following severe pelvic ring fracture: results of a population-based study. Injury.

[2] Blackmore CC, Cummings P, Jurkovich GJ (2006). Predicting major haemmorhage in patients with pelvic fracture. J Trauma.

[3] Cryer HM, Miller FB, Evers BM (1988). Pelvic fracture classification: correlation with hemorrhage. J Trauma.

[4] Chesser TJ, Cross AM, Ward AJ (2012). The use of pelvic binders in the emergent management of potential pelvic trauma. Injury.

[5] Fu CY, Wu YT, Liao CH (2013). Pelvic circumferential compression devices benefit patients with pelvic fractures who need transfers. Am J Emerg Med.

[6] Bonner TJ, Eardley WG, Newell N (2011). Accurate placement of a pelvic binder improves reduction of unstable fractures of the pelvic ring. J Bone Joint Surg Br.

[7] Jain S, Bleibleh S, Marciniak J (2013). A national survey of United Kingdom trauma units on the use of pelvic binders. Int Orthop.

[8] Burgess AR, Eastridge BJ, Young JW (1990). Pelvic ring disruptions: effective classification system and treatment protocols. J Trauma.

[9] Marsh JL, Slongo TF, Agel J (2007). Fracture and dislocation classification compendium - 2007: Orthopaedic Trauma Association classification, database and outcomes committee. J Orthop Trauma.

[10] Croce MA, Magnotti LJ, Savage SA (2007). Emergent pelvic fixation in patients with exsanguinating pelvic fractures. J Am Coll Surg.

[11] Krieg JC, Mohr M, Ellis TJ (2005). Emergent stabilization of pelvic ring injuries by controlled circumferential compression: a clinical trial. J Trauma.

[12] American College of Surgeons (1997). Advanced Trauma Life Support for doctors, ATLS. Instructor course manual.

[13] Ghaemmaghami V, Sperry J, Gunst M (2007). Effects of early use of external pelvic compression on transfusion requirements and mortality in pelvic fractures. Am J Surg.

[14] Spanjersberg WR, Knops SP, Schep NW (2009). Effectiveness and complications of pelvic circumferential compression devices in patients with unstable pelvic fractures: a systematic review of literature. Injury.

[15] Qureshi A, Mcgee A, Cooper JP (2005). Reduction of the posterior pelvic ring by non-invasive stabilisation: a report of two cases. Emerg Med J.

[16] Berg EE, Chebuhar C, Bell RM (1996). Pelvic trauma imaging: a blinded comparison of computed tomography and roentgenograms. J Trauma.

[17] Swartz J, Vaidya R, Hudson I (2015). The Effect of Pelvic Binder Placement on OTA Classification of Pelvic Ring Injuries using Computed Tomography. Does it Mask the Injury?. J Orthop Trauma.

[18] Clements J1, Jeavons R1, White C1 (2015). The Concealment of Significant Pelvic Injuries on Computed Tomography Evaluation by Pelvic Compression Devices. J Emerg Med.

